# Analysis of the Mechanisms of Action of Naphthoquinone-Based Anti-Acute Myeloid Leukemia Chemotherapeutics

**DOI:** 10.3390/molecules24173121

**Published:** 2019-08-28

**Authors:** Michelle H. Lee, Rena G. Lapidus, Dana Ferraris, Ashkan Emadi

**Affiliations:** 1 Department of Medicine Division of Hematology/Oncology University of Maryland School of Medicine, Baltimore, MD 21201, USA; 2 Marlene and Stewart Greenebaum Comprehensive Cancer Center, University of Maryland School of Medicine, 22 South Greene Street, Baltimore, MD 21201, USA; 3 Department of Chemistry, McDaniel College, 2 College Hill, Westminster, MD 21157, USA

**Keywords:** acute myeloid leukemia (AML), naphthoquinone, reactive oxygen species (ROS), apoptosis, mechanism of action

## Abstract

Acute myeloid leukemia (AML) is a neoplastic disorder resulting from clonal proliferation of poorly differentiated immature myeloid cells. Distinct genetic and epigenetic aberrations are key features of AML that account for its variable response to standard therapy. Irrespective of their oncogenic mutations, AML cells produce elevated levels of reactive oxygen species (ROS). They also alter expression and activity of antioxidant enzymes to promote cell proliferation and survival. Subsequently, selective targeting of redox homeostasis in a molecularly heterogeneous disease, such as AML, has been an appealing approach in the development of novel anti-leukemic chemotherapeutics. Naphthoquinones are able to undergo redox cycling and generate ROS in cancer cells, which have made them excellent candidates for testing against AML cells. In addition to inducing oxidative imbalance in AML cells, depending on their structure, naphthoquinones negatively affect other cellular apparatus causing neoplastic cell death. Here we provide an overview of the anti-AML activities of naphthoquinone derivatives, as well as analysis of their mechanism of action, including induction of reduction-oxidation imbalance, alteration in mitochondrial transmembrane potential, Bcl-2 modulation, initiation of DNA damage, and modulation of MAPK and STAT3 activity, alterations in the unfolded protein response and translocation of FOX-related transcription factors to the nucleus.

## 1. Introduction

### 1.1. Chemistry of Naphthoquinones

Naphthoquinones (C_10_H_6_O_2_) are oxidized naphthalenes (C_10_H_8_), and similar to other quinones, such as benzoquinone and anthraquinone, as they possess a conjugated electron system that can participate in chemical reactions transporting electrons to other molecules [1]. These reactions can result in the generation of free radicals, including highly active oxygen radicals called reactive oxygen species (ROS) [1]. Production of ROS occurs when quinones are reduced to semiquinones and subsequently to hydroquinones by one- or two-electron reductions via different enzymes [2,3,4]. While ROS, at low and highly regulated intracellular levels, contribute to normal cellular signaling function; excess ROS plays an important role in damaging cellular components, including proteins, lipids and nucleic acids [5]. Agents with similar structures to naphthoquinones have been approved for use by the FDA. For example, Mitomycin, a benzoquinone, is used in the clinic for intravesical therapy after transurethral resection of bladder tumor for non-invasive (stage 0) or minimally invasive (stage I) bladder cancers, and in combination with 5-fluorouracil (5-FU) and radiation for treatment of stage I-III anal cancer [6,7]. Anthraquinones, such as daunorubicin, idarubicin, doxorubicin, epirubicin and mitoxantrone are commonly used for the treatment of many hematologic and solid neoplasms [8,9].

Natural and synthetic, oligomeric substituted and heterocyclic naphthoquinone derivatives have shown promising anti-neoplastic activities against epithelial, mesenchymal and hematopoietic malignancies [2,10,11,12]. Notwithstanding fifty years of basic, translational, and clinical studies, the mechanism by which naphthoquinone-containing agents induce cancer cell death remains somewhat unclear. The apparent redox-cycling and electrophilic properties of naphthoquinones culminating in ROS generation and induction of apoptosis have subjugated the field of mechanistic investigations of these compounds [13,14]. Nevertheless, several other mechanisms, including covalent alkylation of DNA, DNA intercalation, inhibition of topoisomerase II, epigenetic modulation, to name a few, might be significantly contributing to the cause of death in different cancer cells.

### 1.2. Acute Myeloid Leukemia

Acute myeloid leukemia (AML) is a neoplastic disorder characterized by rapidly proliferating myeloblasts in bone marrow, blood and occasionally other organs resulting in a very poor clinical outcome, particularly in elderly and medically unfit patients. The diverse cytogenetics and molecular mutations of myeloblasts among patients and even within one patient makes AML an extremely heterogeneous blood cancer [15,16]. Irrespective of their genetic characteristics, AML cells generate excess ROS [17]. In turn, these cells have prominent antioxidant machinery to mitigate the effects of additional ROS and maintain cellular oxidative states compatible with cell survival [17,18]. With this in mind, it was hypothesized that naphthoquinone-induced ROS augmentation in myeloblasts could cause an imbalance to the already stressed redox balance, overwhelming the ROS buffering capacity of the AML cells and cause cell death [13]. However, depending on the chemical structures of the naphthoquinone, certain derivatives might negatively affect other cellular machinery involved in the initiation and propagation of neoplastic phenomenon seen in AML. In this article, we aimed to analyze the entire publically available mechanism of actions of naphthoquinone-based anti-AML chemotherapeutics. We believe this will help chemists to synthesize new generations of anti-cancer naphthoquinones and also assist biologists and clinicians to design naphthoquinone-based combination chemotherapies rationally.

## 2. Results

### 2.1. Anti-Proliferative Activity 

Table 1 shows the naphthoquinone derivatives that have been reported to have anti-AML activity. Mono- or di-substituted monomeric naphthoquinones, including menadione [19], juglone [20], lawson [21], glycinyl-1,4-naphthoquinone [22], plumbagin [23,24,25,26,27], lapachol (and nor-lapachol) [21,28,29], atovaquone [30,31], ramentaceone [32], cordiaquinone J [33], and TW-92 [34] showed anti-AML activity in different AML cell lines and primary cells from AML patients with wide IC_50_s ranging from 0.6 to 100 micromolar (μM), Table 1. Atovaquone was found to have an additive effect when combined with standard induction chemotherapy (cytarabine and daunorubicin) in AML cell lines [31]. Multi-substituted naphthoquinones, including shikonin [35,36,37,38,39] and its derivative SH-7 [40], and other dihydroxy or dimethoxy 1,4-naphthoquinones [41,42], compared with mono- or di-substituted naphthoquinones, demonstrated superior in vitro activity against AML cells with IC_50_s ranging 0.1–4 μM. Heterocyclic monomeric naphthoquinones included furanonaphthoquinones FNQ3 [43] and FN6-one [44], β-lapachone [45,46,47,48,49] and nor-β-lapachone [45,46,50,51], dunnione [47], and pterocarpanquinone LQB-118 [28,52]. FNQ3 was significantly more effective than low dose cytarabine in reducing cell viability (*p* < 0.001) and combining the two drugs led to an even greater reduction in cell viability in NB4 and U937 cells (*p* < 0.01) [43].

By inhibiting HIV integrase, the hydroxylated dimeric naphthoquinones were originally synthesized as anti-HIV agents [53,54]. Redox modulating ability of these compounds resulted in studies to determine their potency against AML cells, as well as their therapeutic indices in relation to normal hematopoietic stem cells [4,55]. The IC_50_ values (μM) of 3-bromo-3′-hydroxyl-dimeric 1,4-naphthoquinone (BiQ1) against AML cell lines MOLM-14, THP1, and one primary AML cells from patients, as well as normal bone marrow cells were 5.5 ± 0.8, 4.2 ± 1.9, 0.4 and 14.5, respectively [55]. Bis-aziridinyl dimeric naphthoquinone containing two nitrogen mustard alkylating groups was synthesized to improve the potency and bioavailability of dimeric naphthoquinones [56]. This compound showed a potent anti-leukemic activity (IC_50_ range 0.18–2 μM) against three AML cell lines and four primary AML cells from patients [56]. Of note, the patient-derived AML cells had a heterogeneous cytogenetic and molecular mutation profile. In addition to inducing a decrease in cell survival and viability, exposure to the bis-aziridinyl dimeric naphthoquinone at concentrations relative to the respective IC_50_ values for 24 h resulted in a marked reduction in clonogenic activity—an in vitro assay to assess the capability of a single AML cell in the population to generate a colony—the quintessential anti-cancer in vitro assay [56]. Dimeric naphthoquinones had favorable therapeutic indices as demonstrated by their more potent IC_50_ values in AML cells compared to normal hematopoietic cells.

### 2.2. In Vivo Tolerability and Efficacy Studies

A few studies tested the tolerability and safety of naphthoquinone derivatives in vivo as wells as their anti-AML activity [4,24,35,40,56]. Plumbagin dosed 2 mg/kg via intraperitoneal injection, IP) daily for three weeks significantly reduced tumor volume in NB4 tumor xenograft in NOD/SCID mice; 153 mm^3^ in plumbagin versus 194 mm^3^ in control after one week of treatment and approximately 65% tumor volume reduction at the completion of the study [24]. In the same study using the same xenograft model, tumors of mice treated with doxorubicin (1 mg/kg thrice weekly) demonstrated greater regression in tumor volume than those treated with plumbagin (*p* < 0.05). However, doxorubicin at the doses tested was toxic to heart and liver (pathologic findings), whereas plumbagin treated mice did not show any obvious tissue changes.

Our group tested tolerability of a different naphthoquinone containing a compound called BiQ1 in mice dosed via IP and subcutaneous (SC) injections [4]. Mice tolerated eight days of IP injections dosed at 10 mg/kg with no overt weight loss or physical complications. However, mice were unable to ambulate, eat or drink with just two doses of 25 mg/kg IP injections. Interestingly, mice were able to tolerate SC doses of 25 mg/kg with the only overt side effect being irritation at the injection site. The poorer tolerability of IP versus SC at higher dose might be related to the acidity of BiQ1 injected in intra-abdominal cavity of mice causing peritonitis. 

Carter-Cooper et al. reported that 3,3′-bis-aziridinyl dimeric naphthoquinone was well tolerated by NOD SCID gamma (NSG) mice after five consecutive days of 5, 10, and 15 mg/kg (IP) without significant weight loss [56]. Finally, Nestal de Moraes et al. reported that naphthoquinone derivatives may be safe against normal bone marrow-derived cells by testing compound LQB-118 IP in mice with normal bone marrow cells [52]. 

Atovaquone is an FDA-approved, anti-microbial drug that has been well tolerated, even in immunosuppressed patients with organ dysfunction. In a retrospective study of adult AML patients who underwent allogeneic hematopoietic stem cell transplant (HSCT), patients who received atovaquone for longer duration had lower rates of AML relapse over three years compared to those with lower atovaquone exposure (13% versus 23%, *p* = 0.037) [30]. *In vivo*, female mice treated with atovaquone 200 mg/kg daily demonstrated decreased disease burden compared to the control mice [31]. Furthermore, prolonged atovaquone survival of male xenografted mice compared to the control mice (median survival of 63 days in control mice versus only one mouse succumbing to disease by day 70 in the atovaquone group, *p* = 0.0048). 

### 2.3. Mechanisms of Anti-AML Action

#### 2.3.1. Induction of Apoptosis 

The primary mechanism by which naphthoquinones reduce AML cell survival is via apoptosis. A dose- and time-dependent increase in apoptosis was observed with several naphthoquinone-based compounds. Induction of apoptosis rather than necrosis as the cause of cell death was evident by changes observed in cell morphology, cell cycle progression, and caspase activation. Interestingly, 5,8-dihydroxy-2-(1-hydroxy-2-nitroethyl)naphthalene-1,4-dione [41] and LQB-118 [52] induced apoptosis without significant changes observed in cell cycle. The anti-AML activity of these naphthoquinones appeared to be mediated through a combination of ROS enhancement and topoisomerase inhibition that ultimately led to DNA damage and cell death. 

#### 2.3.2. Alteration in Mitochondrial Membrane Potential 

Naphthoquinone derivatives reduce mitochondrial membrane potential (MMP) by the opening of the mitochondrial permeability transition pore resulting in cytochrome C release into the cytosol and activating caspase-3; key features of the intrinsic pathway of apoptosis [57,58]. Hallak et al. reported that TW-74, a chloronaphthoquinone with a methyl group at the meta position, effectively killed U937 myeloid leukemia cells via early reduction of MMP, cytochrome C release and caspase activation [59]. We reported that hydroxylated dimeric naphthoquinone BiQ1 effectively inhibited induced depolarization of the mitochondrial transmembrane potential (ΔΨm) as measured by flow cytometry with MitoPotential Red [4].

#### 2.3.3. Reduction-Oxidation Imbalance 

ROS are derived from the metabolism of oxygen, and they normally exist in balance with antioxidants in all cells. Oxidative stress occurs when this balance is disrupted, due to excess production of ROS and/or antioxidant diminution. Generation of ROS and the consequent increase in oxidative stress has proven to be fundamental in the cytotoxic activity of naphthoquinone derived compounds. Given the high proliferative index, most AML cells, compared with non-malignant cells, have a higher level of endogenous ROS, lower activities in respiratory chain complex and lower spare reserve capacity [60,61]. This phenomenon, as well as lower compensatory reserves that handle additional oxygen radicals [13,60], make AML cells particularly sensitive to oxidative stress [62]. Several studies have demonstrated that the presence of antioxidants significantly attenuates mitochondrial-dependent apoptosis and cell cycle arrest in naphthoquinone-treated tumor cells [24,34,63]. While antioxidants, such as N-acetyl cysteine (NAC) may serve to lessen naphthoquinone cytotoxicity by neutralizing ROS through increased glutathione production, they also could exert their actions by chemically interacting directly with naphthoquinones or their derivatives to reduce their cytotoxicity [1,64,65]. 

Much evidence exists in the literature linking redox imbalance and anti-AML activity. Treatment of leukemic cell lines with bis-aziridinyl dimeric naphthoquinone resulted in a dose-dependent increase in intracellular production of ROS within two hours of exposure [56]. Plumbagin similarly demonstrated increased intracellular ROS in NB4 cells that peaked after 1.5 h of exposure with a decline after two hours [66]. Xu and colleagues further demonstrated that ROS production was significantly increased in NB4 cells treated with plumbagin compared to healthy control cells, indicative of selective toxicity against neoplastic cells [24]. The chloro-amino-phenyl naphthoquinone TW-92 induced apoptosis in U937 cells, which was preceded by accumulation of intracellular hydrogen peroxide that was abolished by an inhibitor of NADPH oxidase [34]. U937 cells treated with TW-92 versus untreated cells showed a rapid decline in endogenous glutathione levels (65%) [34]. This depletion of glutathione may have contributed to the intracellular accumulation of hydrogen peroxide. Chau et al. compared intracellular H_2_O_2_ concentration in HL-60 cells treated with β-lapachone (a naphthoquinone) and camptothecin (a non-quinone topoisomerase inhibitor) [67]. The study found a seven-fold increase in H_2_O_2_ production in HL-60 cells treated with β-lapachone compared to a less than two-fold increase in cells treated with camptothecin. This increase in H_2_O_2_ was markedly reduced in the presence of NAC, which corresponded with reduced β-lapachone-induced apoptosis as evidenced by reduced DNA fragmentation and sub-G1 hypodiploid cells. These results suggest that intracellular increase in H_2_O_2_ may be one mechanism by which naphthoquinones induce cell death. 

#### 2.3.4. DNA Damage 

Quinones are able to induce DNA damage, as shown by the increase in histone H2Ax phosphorylation, a step which always follows double-stranded DNA breaks to activate apoptosis [68]. Quinones have also been shown to exert antitumor effects by inhibiting nuclear enzyme topoisomerases I and II [69]. Topoisomerase mediated DNA damage of leukemic cells was observed in the activity of a synthetic naphthoquinone derivative of alkannin and shikonin against HL-60 cells [41]. β-Lapachone (3,4-dihydro-2,2-dimethyl-2H-naphtho[1,2-b]pyran-5,6-dione) is also a topoisomerase I inhibitor [63], by the proposed mechanism of locking the enzyme onto the DNA and blocking replication fork movement [48]. 

Shikonin has been shown to inhibit both topoisomerases I and II in different cancer cells [70,71,72]. To overcome the poor solubility of Shikonin, its derivative SH-7 was synthesized [40]. SH-7 displayed significant cytotoxicity against HL-60 cells with IC_50_ of 2 μM and showed significant inhibition of topoisomerase II via stabilization of the topoisomerase II-DNA cleavable complex [40]. Bey et al. suggested that the anti-tumor activity of topoisomerases inhibitors may be attributed to the overexpression of the enzymes and associated deficiency of protective mechanism against DNA damage in cancer cells or to the increase in topoisomerase bioactivation by the NAD(P)H:quinone oxidoreductase (NQO1) [73].

#### 2.3.5. Bcl-2 Modulation 

Bcl-2 family of proteins are integral to the mitochondria-dependent pathway of apoptosis [74]. Cell death is regulated by a complex interaction between the pro-apoptotic and pro-survival members of the Bcl-2 family. Pro-apoptotic members of the Bcl-2 family, such as Bax or Bak translocate from the cytosol to the mitochondria, leading to the release of cytochrome C into the cytosol [75]. Anti-apoptotic proteins, Bcl-2 [76] and Mcl-1 [77] have been studied extensively, given their ability to induce chemo-resistance in different cancers, including AML. Venetoclax, a Bcl-2 inhibitor, in combination with azacitidine or decitabine or low-dose cytarabine is approved (conditionally) by the US Food and Drug Administration (FDA) for treatment of adult patients with newly-diagnosed AML who are age 75 years or older, or who have comorbidities that preclude use of intensive induction chemotherapy [78].

The antitumor activities of naphthoquinones may be mediated by their ability to up- or down-regulate the proteins in the Bcl-2 family [4,79,80]. Shikonin down-regulated antiapoptotic proteins, such as Bcl-2 and Bcl-XL in AML cells and induced apoptosis [79]. Plumbagin-treated NB4 cells had increased expression of Bax and Bak with a decrease in Bcl-XL within 8 h of treatment [24]. TW-92 decreased expression of Mcl-1, increased Bax expression without change in expression of Bcl-2 and induced apoptosis in U937 cells [34]. Chau and colleagues reported ectopic overexpression of Bcl-2 in HL-60 cells acquired resistant phenotype to β-lapachone with decreased intracellular H_2_O_2_ [67].

#### 2.3.6. MAPK Pathway 

Mitogen-activated protein kinase (MAPK) pathways are involved in the regulation of cellular stress response by transducing extracellular signals to the cell nucleus to impact transcription factors important for cell proliferation, differentiation, survival, and apoptosis [81]. Constitutive activation of the MAPK cascades drives the oncogenic transformation of normal fibroblasts and is commonly detected in cancers. Inappropriate activation of these pathways may play a role in leukemic transformation of myeloid cells and their ability to proliferate and escape programmed cell death [82]. Several naphthoquinones have shown the ability to impact the MAPK pathways to induce apoptosis in leukemic cells [34,37], which may be a promising therapeutic strategy for AML. In the shikonin and shikonin derivative study [37], these agent’s influence on MAPK and AKT signaling cascades led to apoptosis of AML cells in vitro by direct interaction, and down-regulation of c-MYC, a transcription factor integral to cell cycle regulation and proliferation [37]. More investigations are warranted on the role of MAPK cascades on naphthoquinone-induced apoptosis as it has been established that MAPK cascades converge to the mitochondria and promote mitochondrial-induced apoptosis by complex interplay with Bcl-2 family [83]. 

#### 2.3.7. STAT3 Inhibition

Signal transducer and activator of transcription 3 (STAT3) is an oncogenic transcription factor that is often dysregulated in AML and can be used as a valid target for AML treatment [84]. Atovaquone is an anti-microbial drug that has recently been discovered to have antileukemic efficacy in vitro and in vivo [31]. Xiang and colleagues found that atovaquone diminishes the expression of gp130, a key protein involved in STAT3 activation which in turn potently inhibited STAT3 signaling from interleukin-6 (IL-6), a critical survival factor in AML [30]. The reduction of STAT3 activation consequently led to the reduction of STAT3 target genes mediating cell survival and proliferation culminating in apoptosis. 

#### 2.3.8. Unfolded Protein Response Modulation

Unfolded protein responses (UPR) is a mechanism by which cells are able to combat adverse effects of protein accumulation in the endoplasmic reticulum (ER) following ER stress by inducing activation of this pro-apoptotic pathway [85]. UPR plays an important role in chemo-resistance in cancer cells, including leukemia cells [86,87]. ERP57, a key protein situated on the ER, is involved in the proper folding of newly synthesized polypeptides, and is overexpressed in AML cells compared to healthy cells [36]. Overexpression of ERP57 significantly reduced Shikonin-induced apoptosis, though its expression is downregulated by Shikonin itself [36]. 

#### 2.3.9. Forkhead Box (Fox) Proteins 

Fox proteins are a family of transcription factors that regulate the expression of genes involved in cell proliferation, growth, and differentiation [88]. They recognize DNA breaks and initiate repair or excision by binding to the Forkhead response element region (FHRE). Members of Fox class M and O have been identified as essential components of oncogenic and tumor suppressive pathways and are often deregulated in leukemia. 

FOXO3A is a tumor suppressor transcription factor that is a member of the FoxO subfamily [89]. High levels of phosphorylated FOXO3A was identified as an independent, poor prognostic factor in AML and was associated with increased leukemic cell proliferation, drug resistance, and shorter duration of remission [90]. Phosphorylation leads to cytoplasmic localization and inactivation of FOXO3A, which leads to the proliferation of leukemic cells in AML. Treatment of HL-60 cells with naphthoquinone LQB-118 resulted in FOXO3A nuclear translocation with upregulation of Bim, a pro-apoptotic member of the Bcl-2 family [52]. In contrast, U937 cells treated with LQB-118 had FOXO3A nuclear exclusion and Bim downregulation [52] suggesting that LQB-118 promotes differential modulation of FOXO3A localization in different AML subtypes. 

Fox protein M1 (FOXM1) is a regulator of cell cycle progression and was found to be abnormally expressed in AML blasts [91]. LQB-118-treated U937 cells had progressively decreasing FOXM1 expression, with concurrent downregulation of Survivin [52]. Of note, Survivin is an anti-apoptotic protein that is correlated with poor prognosis in AML and is known to be regulated by FOXM1 [92]. 

## 3. Methods and Materials 

### 3.1. Search Strategy and Inclusion Criteria

An electronic search was conducted on the MEDLINE database for articles published up to 7 August 2018, using the combined search terms *acute*, *leukemia*, and *naphthoquinone*. Seventy-eight articles were preliminarily identified. Studies that investigated the activity of naphthoquinone derivatives against AML were selectively reviewed. Twenty-one publications reported that anti-AML efficacy, in addition to at least one proposed mechanism of action of the studied naphthoquinone(s) were included for this analysis. The reference lists of these selected articles were also examined to include any relevant publications not captured in the initial search. In this report, we will not compare or comment on the study designs of the included articles. Additionally, in studies that synthesized and analyzed multiple naphthoquinone derivatives with the same core structure, only the compound with the strongest activity against AML cells, as determined by their IC_50_ (i.e., concentration that decreases viable cell numbers by 50%) values, were included for discussion. 

### 3.2. Cell Cultures 

In the included studies, in vitro activities of the naphthoquinone derivatives were examined in AML cell lines, and myeloblasts isolated from peripheral blood (PB) and bone marrow (BM) of AML patients. Often, the cytotoxicity of the compounds was studied in normal human cell lines and primary cells from healthy donors as comparators. Just a few in vivo studies have been conducted to examine the safety and tolerability of naphthoquinone-based agents. 

### 3.3. Cell Proliferation and Cell Survival Assays 

Inhibition of cell growth was assessed by measuring cell metabolic activity by MTT-like assays, including 3-(4,5-dimethylthiazol-2-yl)-2,5-diphenyltetrazolium bromide (MTT), (3-(4,5-dimethylthiazol-2-yl)-5-(3-carboxymethoxyphenyl)-2-(4-sulfophenyl)-2H-tetrazolium) (XTT), and water-soluble tetrazolium salts (WST) assays. Cell viability was determined using trypan blue exclusion. 

### 3.4. Analysis of Apoptosis 

Apoptosis was measured using the Annexin V flow cytometric kits, or acridine orange/ethidium bromide stains. Means of cell death were further evaluated with cell cycle progression (propidium iodide), mitochondrial membrane potential, Western Blot analyses, nuclei staining, or caspase activation. 

## 4. Conclusions

The ability of naphthoquinone analogs to affect multiple steps in the apoptotic pathway and cellular signaling pathways play a role in their anti-AML activity. The simple mono- and di-substituted naphthoquinones have the ability to inhibit cell growth of various AML cell lines at low micromolar concentrations with the most potent derivatives being the 5-hydroxy-substituted derivative plumbagin (0.6–1.4 μM). Consequently, plumbagin also increases intracellular ROS and decreases expression of Bcl-2, indicating that the increased potency may be due to multiple mechanisms of action. From the multi-substituted naphthoquinones, this same pattern is observed with the shikonin derivatives demonstrating low micromolar (shikonin and SH-7) and in one case, (5,8-dihydroxy-2-(1-hydroxy-2-nitroethyl)-1,4-naphthoquinone, 0.14 μM) sub-micromolar potency against AML cell lines. Most likely multiple mechanisms of action for these derivatives provides the robust activity, including inhibition of topoisomerase, down-regulation of Bcl-2/BclxL, and down regulation of c-Myc and maybe the unfolded protein response. Thus, depending on the oxidoreductases or other bases for altered electron donation, present in AML cells and a few other neoplastic cells, the electron-accepting potential of quinones could in principle be “tuned” to yield selective cytotoxicity for cells with a particular “redox” environment, allowing initiation of a cascade of electron transport only in malignant cells, with dysregulated redox state, and not in normal cells, with the consequent increase in ROS producing selective cell killing. The dimeric naphthoquinones represent some of the most potent compounds against AML cell lines, and they have the ability to increase intracellular ROS like plumbagin.

## Figures and Tables

**Table 1:** Anti-acute myeloid leukemia (AML) naphthoquinones.

Structure	AML Cell Lines	IC_50_ (μM)	In Vivo	References
***Mono- or di-substituted monomeric naphthoquinones***				
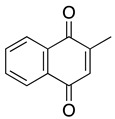 **Menadione** **2-methyl-1,4-naphthoquinone**	HL-60, U937	Not reported directly	Not tested	Yeo HS, et al. 2012
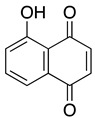 **Juglone** **5-hydroxyl-1,4-naphthoquinone**	HL-60	8	Not tested	Xu HL, et al., 2010
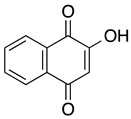 **Lawsone** **2-hydroxyl-1,4-naphthoquinone**	HL-60	>50	Not tested	Esteves-Souza A, et al., 2008
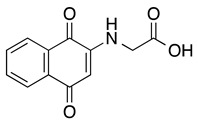 **2-glycinyl-1,4-naphthoquinone**	HL-60	3	Not tested	de Moraes TA, et al., 2014
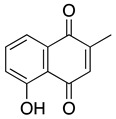 **Plumbagin** **5-hydroxy-2-methyl-1,4-naphthoquinone**	Kasumi-1, HL-60, U937	0.6–1.4	Activity: Tumor volume reduction Tolerability: No significant weight loss, tissue damage or behavior change (Xu KH, et al.)	Kawiak A, et al., 2007 Xu KH, et al., 2010 Gaascht F, et al., 2014 Kong X, et al., 2017 Zhang J, et al., 2017
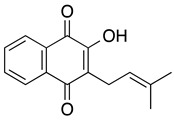 **Lapachol** **2-hydroxy-3-(3-methyl-2-butenyl)-1,4-naphthoquinone**	Kasumi-1 Primary cells from patients HL-60	Not reported directly >50	Not tested	de Souza Reis FR, et al., 2013 Esteves-Souza A, et al., 2008
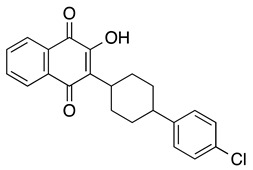 **Atovaquone** ***trans*** **-2-[4-(4-chlorophenyl)cyclohexyl]-3-hydroxy-1,4-naphthalenedione**	MOLM13, MV4-11, THP-1, NB4, Kasumi-1, HL-60, KG1, HEL	13.7–98.9	Decreased diseased burden and prolonged survival in the treatment group compared to the control group (Stevens AM, et al.)	Xiang M, et al., 2016 Stevens AM, et al., 2017
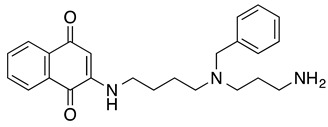 **2-((4-((3-aminopropyl)(benzyl)amino)butyl) amino)naphthalene-1,4-dione**	HL-60	10.5	Not tested	Esteves-Souza A, et al., 2008
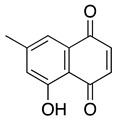 **Ramentaceone** **7-Methyljuglone**	HL-60	8.8	Not tested	Kawiak A, et al., 2012
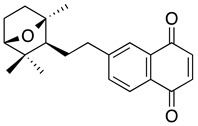 **Cordiaquinone J**	HL-60	2.7	Not tested	Marinho-Filho JD., et al., 2010
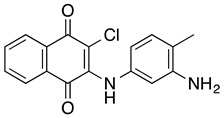 **TW-92** **2-chloro-3-(3-amino-4-methyl-phenylamino)-1,4-naphthoquinone**	U937, peripheral blood mononuclear cells from AML patients	3.2	Not tested	Hallak M, et al., 2009
***Multi-substituted monomeric naphthoquinones***				
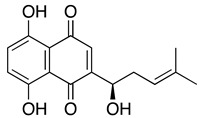 **Shikonin** **5,8-dihydroxy-2[(1*R*)-hydroxy-4-methylpent-3-en-1-yl]-3-methyl-1,4-naphthoquinone**	HL-60, U937, primary AML cells	3.8	Prolonged survival observed in the treatment group compared to the control group (Yang H, et al.)	Yang H, et al., 2009 Zhang B, et al. 2012 Zhao Q, et al., 2015 Trivedi, et al., 2016
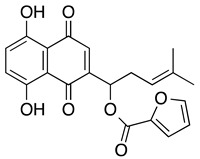 **SH-7** **1-(1,4-dihydro-5,8-dihydroxy-1,4-dioxonaphthalen-2-yl)-4-methylpent-3-enylfuran-2-caroxylate**	HL-60	2	Not tested	Yang F, et al., 2006
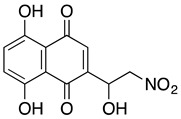 **5,8-dihydroxy-2-(1-hydroxy-2-nitroethyl)-1,4-naphthoquinone**	HL-60	0.14	Not tested	Beretta GL, et al., 2017
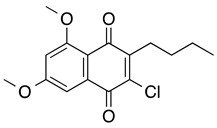 **3-butyl-2-chloro-5,7-dimethoxy-1,4-naphthoquinone**	HL-60	3.8	Not tested	Li K, et al., 2018
***Heterocyclic monomeric naphthoquinones***				
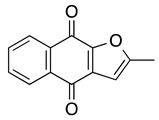 **FNQ3 (Furanonaphthquinone)** **2-Methylnaphtho[2–*b*]furan-4,9-dione**	HL60, NB-4, U937, THP1, primary AML cells	5.9–8.2	Not tested	Desmond JC, et al., 2005
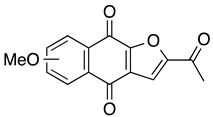 **FN6-one (Furonaphthoquinone)**	HL-60, U937	0.87–3.0	Not tested	Inagaki R, et al., 2013
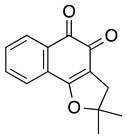 **Nor-β-lapachone**	HL-60, KG1, K562	0.4–17.2	Not tested	da Silva EN Jr, et al. 2007, 2010 Cavalcanti, et al., 2011 Araújo AJ, et al., 2012 Cardoso, et al., 2014
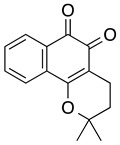 **β-lapachone** **3,4-dihydro-2,2-dimethyl-2H-naphtho[1–b]pyran-5,6-dione**	HL-60	7.1	Not tested	Planchon SM, et al., 1995, 1999 da Silva EN Jr, et al. 2007, 2010 Inagaki, et al., 2015
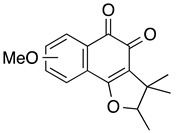 **Dunnione**	HL-60	0.9	Not tested	Inagaki, et al., 2015
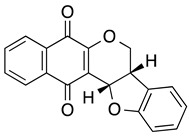 **LQB-118 (pterocarpanquinone)**	HL60, U937, Kasumi-1	6–9	Non-cytotoxic to normal BM cells in healthy control mice.	de Souza Reis, et al., 2013, Nestal De Moraes, et al., 2014
***Dimeric naphthoquinones***				
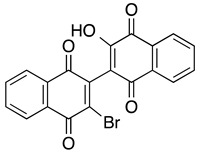 **3-bromo-3′-hydroxyl-dimeric 1,4-naphthoquinone**	MOLM-14, THP-1, primary AML cells	0.36–8.5	Tested for tolerability and side effects with IP and SC injections.	Lapidus, et al., 2016
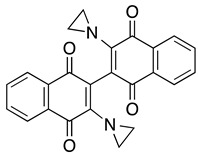 **3,3′-bis-aziridinyl-dimeric 1,4-naphthoquinone**	MOLM-14, THP-1, primary AML cells	0.18–2	Tested for tolerability with IP injections.	Carter-Cooper, et al., 2017

BM = bone marrow, IP = intraperitoneally, SC = subcutaneously.
